# Exploring reasons why South African dental therapists are leaving their profession: A theory-informed qualitative study

**DOI:** 10.1371/journal.pone.0293039

**Published:** 2023-10-26

**Authors:** Pumla Pamella Sodo, Yolanda Malele-Kolisa, Aneesa Moola, Veerasamy Yengopal, Simon Nemutandani, Sara Jewett

**Affiliations:** 1 School of Clinical Medicine, University of the Witwatersrand, Johannesburg, South Africa; 2 School of Oral Health Sciences, University of the Witwatersrand, Johannesburg, South Africa; 3 Faculty of Dentistry, University of Western Cape, Cape Town, South Africa; 4 Research and Innovation Directorate, Sefako Makgatho Health Sciences University, Pretoria, South Africa; 5 School of Public Health, University of the Witwatersrand, Johannesburg, South Africa; Shahid Beheshti University of Medical Sciences School of Dentistry, ISLAMIC REPUBLIC OF IRAN

## Abstract

**Background:**

Dental therapy is a category of mid-level oral health professional that was introduced to address inequities in oral health service provision in South Africa within a constrained human resource for health context. However, low numbers of registered dental therapists and attrition threaten this strategy.

**Aim:**

This study explored reasons for this attrition, building on the Hertzberg Two-Factor Theory.

**Methods:**

Through a qualitative exploratory study design, in-depth interviews were conducted with former dental therapists to explore their reasons for leaving the profession. They were recruited using snowball sampling. All interviews were audio recorded, transcribed verbatim, and coded in NVIVO12. A team of researchers applied thematic analysis to agree on themes and sub-themes, guided by Hertzberg’s ideas of intrinsic and extrinsic factors.

**Findings:**

All 14 former dental therapists interviewed expressed their passion for the profession, even though their motivations to join the profession varied. Many of their reasons for leaving aligned with extrinsic and intrinsic factors defined in Hertzberg’s Two-Factor Theory. However, they also spoke about a desire for a professional identity that was recognized and respected within the oral health profession, health system, and communities. This is a novel study contribution.

**Conclusion:**

Dental therapist attrition in South Africa is mainly caused by job dissatisfaction and motivation issues resulting from health system level factors. While the Hertzberg Two-Factor Theory helped identify extrinsic and intrinsic factors at an individual level, we used the Human Resources for Health System Development Analytical Framework to identify solutions for dental therapist production, deployment, and retention. Addressing these issues will enhance retention and accessibility to oral health services in the country.

## Background

Dental therapy is a category of mid-level oral health professionals that was introduced to the South African health system in 1977 to meet the population’s oral health needs and to address inequities in oral health service provision [1–4]. The scope of dental therapy focuses on the holistic care of patients, which ranges from the prevention of oral disease and oral health promotion to the alleviation of pain, sepsis, and other diseases such as gingivitis, periodontitis, and dental caries [5]. Dental therapists’ function in the fields of preventive, promotive, and intra-oral restoration of dental tissues, at primary and secondary levels. They treat and prevent the most prevalent oral health problems in South Africa, such as dental caries [6]. They are considered to be an economically sustainable mid-level health professional category because the salary of one dentist is equivalent to employing two to three dental therapists [7]. The dental therapy profession falls within the World Health Organization’s (WHO) Global Strategy on Human Resources for Health (HRH): Workforce 2030, which acknowledges mid-level health workers as having a critical role to play across all service delivery priorities [8].

Both globally and within South Africa’s current context, dental therapists can play an important role in addressing the oral health burden. Many scholars have argued that dental therapists could contribute to achieving access to basic oral health services and lowering the burden of untreated oral health diseases, especially among underserved populations [1, 2, 6, 9, 10]. The WHO, in 2023, reported that 44% of the population in the African region suffer from oral diseases [11]; the last national South African oral health survey reported that 80% of children have untreated oral diseases (unmet need) [6]. In addition to a high prevalence of untreated oral health diseases, there is also limited access to basic oral health services. A South African study conducted in the Western Cape reported that only 31.5% of 128 dental facilities offered the full basic treatment package [12]. As set out in the Primary Health Care Norms and Standards document [13], this includes the provision of pain and sepsis treatment, basic fillings, scaling and polishing, as well as disease prevention and oral health promotion.

A major contributing factor to low oral health service coverage is unavailability of dental professionals, especially in remote areas, as well as a lack of equipment [12]. The Western Cape is among the provinces with the highest prevalence of dental caries [6]. However, there was only one dental therapist employed in the public sector in the Western Cape out of the 714 who were registered in SA in 2019 [14]. Only two out of six dental schools in South Africa train dental therapists [14], which raises concerns about production of this cadre of mid-level oral health professionals.

While dental therapy provides a potential way to increase oral health service coverage within a resource constrained context, attrition within the profession poses a potential threat. Dental therapist attrition has been reported globally; a study conducted among Australian dental therapists who were registered over a five-year period (1999–2003), reported that 28% of participants left the profession for reasons such as family, career change, poor salaries, relocation, illness and injury, and stress [15]. Similarly, Sodo and colleagues have reported attrition and underproduction in South Africa [14]. The numbers of registered dental therapists remain low because some choose to leave the profession due to dissatisfaction or to start new careers [3, 16]. A recent 42-year (1977–2019) review of South African dental therapists reported low number of dental therapists being registered with the Health Professions Council of South Africa (HPCSA) annually, with a 40% attrition rate over the review period and a 9% attrition rate for the ten-year period between 2010 and 2019 [14]. Another South African regional study conducted among University of KwaZulu-Natal (UKZN) graduates reported that 26% of dental therapists in their sample left the profession, of whom 19% returned to university to study dentistry, while 7% no longer worked in the dental profession due to job dissatisfaction [3].

There are various reasons that contribute to voluntary attrition. We used the Hertzberg Two-Factor Theory as a loose framework to explore the issue of employee retention in this study. The theory suggests that attrition is influenced by intrinsic (also called motivation) factors and extrinsic (also called hygiene) factors [17]. According to Herzberg, extrinsic factors (such as salary, working conditions, and job security) are essential for preventing dissatisfaction, but they do not directly lead to motivation or job satisfaction [17]. When extrinsic factors are absent or inadequate, employees may experience dissatisfaction and become more prone to leaving the organization. If these factors are not met, employees may feel unappreciated or undervalued, which can contribute to their decision to seek opportunities elsewhere. In other words, if extrinsic factors are not addressed, they may inadvertently increase the risk of attrition. On the other hand, intrinsic factors (such as recognition, challenging work, and opportunities for growth) are crucial for promoting job satisfaction and motivation [17]. When these factors are present, people are more likely to feel fulfilled and engaged in their work, which can reduce the likelihood of attrition. Motivated employees who find their work meaningful and rewarding are more likely to remain within their jobs and contribute.

Although the Herzberg Two-Factor Theory is useful to identify individual-level factors that influence job satisfaction and dissatisfaction in the workplace, it doesn’t explicitly address broader systemic or organizational factors that might affect job satisfaction, such as structural issues within a health system [16]. Therefore, when evaluating and addressing job satisfaction and motivation within a health system or any other organization, it’s necessary to consider using other frameworks, such as Human Resources for Health System Development (HRHSD) framework [18], which we used to explore health system issues.

Various factors have been identified as driving the voluntary attrition of mid-level health professionals and specifically dental therapists. Studies from South Africa and Australia consistently cite the lack of jobs and promotion opportunities, lack of recognition, poor salaries, limited scope, lack of career pathing, poor working conditions, and lack of resources as the main sources of frustration and job dissatisfaction [14, 16, 19]. Several studies have reported that this dissatisfaction led to them leaving the profession [3, 15, 16, 19]. Studies reported that some dental therapists who leave their profession go back to university to study medicine or dentistry, while some ventured into unspecified businesses [3, 16].

The previous studies did not include dental therapists who had left their profession as participants. Therefore, the focus of this study was to investigate the reasons behind the attrition of dental therapists in South Africa. The study sought to gather insights from former dental therapists to understand their perspectives on why they left the profession.

## Methods

### Study design

This study utilized an exploratory qualitative design, which privileged first-hand experiences of dental therapy attrition, guided by the Hertzberg Two-Factor Theory.

### Study setting

The study was conducted in all nine provinces of South Africa, namely, the Eastern Cape, the Free State, Gauteng, KwaZulu-Natal, Limpopo, Mpumalanga, the Northern Cape, North-West, and the Western Cape.

### Study participants

All qualified dental therapists who left the profession were eligible to be included in the study.

### Sampling and recruitment of participants

The study employed a snowball sampling technique to identify participants from the pool of former dental therapists, a process conducted between March 1, 2020, and June 30, 2022. Initial recruitment involved a combination of participants known to the researchers and those identified through the HPCSA database (for those registered as dentists, medical doctors, or dental specialists). The database had information of participants from all nine South African provinces. The first author (PS) searched for the contact details of potential participants from various sources, such as medical pages, and then contacted and invited them to participate in the study. To expand the participant pool, a respondent-driven sampling approach also was employed. Following each interview, PS requested participants to share study information with other former dental therapists in their networks. Interested participants provided their contact details to PS, through their colleagues, for recruitment purposes. This method aimed to create a network or snowball effect, wherein participants took an active role in recruiting others within their professional circles. This approach facilitated the expansion of the sample and allowed for a more diverse range of perspectives to be included in the study.

### Data collection tool

PS developed a semi-structured interview guide for data collection. The interview guide included questions related to participants’ motivation for choosing a dental therapy career, their lived experiences as well as their reasons for leaving the profession. The questions were based on literature and Hertzberg’s Two-Factor Theory. After co-authors reviewed the guide for content validity and clarity, the interview guide was piloted with three former dental therapists who were not included in this study. No changes were made following the pilot.

### Data collection process

Eligible dental therapists who consented to participate were given an option to either be interviewed in person (at their workplace or homes) or virtually (on Zoom or Teams). PS, herself a female dental therapist by training who now works as an academic, conducted semi-structured in-depth interviews (IDIs) to explore the reasons for attrition in the dental therapy profession with former dental therapists (see S1 File). PS is an MPH graduate and a PhD candidate with experience in conducting IDIs. She had no relationship with the participants prior to the study. The interviews lasted between 20–45 minutes and were audio recorded with the participant’s permission. All interviews were conducted in English. Some interviews were conducted in person, and some were virtual due to COVID 19 restrictions and based on participants’ preferences. PS took detailed field notes during data collection that contained the events, conversations and behaviours observed during the IDIs, and her reflections on them. After study participants stopped raising new ideas or experiences s, PS decided to end interviews, as saturation had been achieved.

### Data analysis

Digital recordings of the interviews were transcribed verbatim by a professional service provider. Following transcription, PS listened to all the audio recordings while reading the transcripts to ensure completeness of the data. A team conducted thematic analysis to analyse the data, following the process detailed by Braun and Clarke: through gaining familiarity with the data; generating initial codes or labels; searching for themes or main ideas; reviewing themes or main ideas; defining and naming themes or main ideas; and producing the report [20]. PS was aware of her own bias as a former dental therapist, hence three more researchers who are not dental therapists were involved in the data analysis. The transcripts were read repeatedly for familiarization with the data by four of the authors: PS, YM-K (dental specialist), AM (oral hygienist), and SJ (one of the first author’s supervisors who is a non-dental health professional). Upon familiarizations with the data, three researchers (PS, YM-K, AM) generated initial codes to guide the development of the coding template that was used for identifying the rest of the codes. Coding was conducted until no further codes were identified and the research team felt that the ideas from the interviews were fully covered. Indexing and charting of the data involved merging the codes into patterns of similarities and differences and aligning them to themes and sub-themes. The data were integrated as we assessed the links among data from participants; this was done and finalised in a meeting by the four researchers (PS, SJ, YM-K, and AM).

### Trustworthiness

To ensure trustworthiness, transcripts were shared with some of the participants to give them an opportunity to clarify transcripts There was no feedback received from participants, they agreed with the transcripts. This was part of respondent validation and establishing the credibility of data as recommended by Lindheim [21]. To ensure the dependability of the findings [21], the last author (SJ) cross-checked the entire analysis process; any discrepancies were resolved by the co-authors. All of the authors (PS, SJ, YK, AM, VY and MN) reviewed the final document.

### Findings

A total of 14 IDIs were conducted with former dental therapists, 11 female and 3 males. No other demographic details were recorded. While gendered patterns were considered during analysis, nothing was identified to suggest experiences were different by gender, although the gender of participants is noted in verbatim quotes.

**Fig 1:**
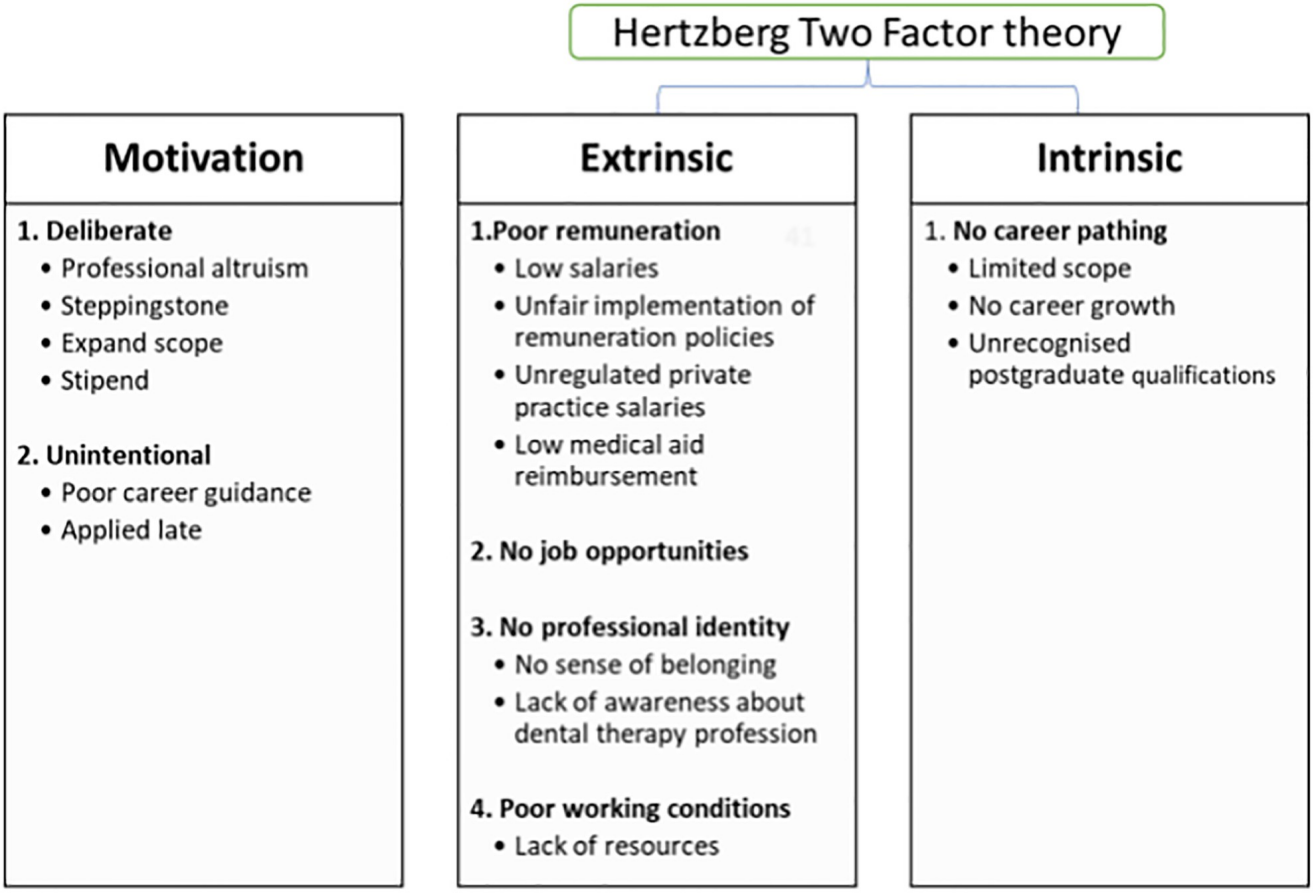
Themes and sub-themes linked to dental therapy job motivation and job dissatisfaction.

Three organising themes (motivation, extrinsic and intrinsic factors) were identified, of which two (extrinsic and intrinsic factors) were deductively drawn from Herzberg’s Two-Factor Theory to categorise the data [17]. The first theme, motivation, refers specifically to what attracted the participants to the dental therapy profession, at the point of training, whereas extrinsic and intrinsic themes were used to identify factors that pushed participants away from the dental therapy profession following training. Using these organising themes enabled categorisation of additional themes and sub-themes, identified inductively from the data. These themes and sub-themes were expressed directly by the (former) dental therapists. Fig 1 summarises the main themes and seven related sub-themes identified from qualitative interviews under each of the three organising themes.

### Themes related to motivation

While some dental therapists made a deliberate decision to enrol in the profession, a key finding was that not all participants wanted to be dental therapists. Some found themselves in the profession unintentionally.

Those who **deliberately** joined the profession, as denoted in our first theme, had several reasons for doing so. The first reason was ***professional altruism***. Some participants saw a gap in service delivery in their communities and took an informed decision to enrol in a dental therapy degree out of their desire to provide beneficial services for their community. One participant said: *“I came to dental therapy because I love it and I knew that I could do something for my people*. *So I knew if I could do dental therapy*, *then I would be able to serve my community*.*” (DT 8*, *female)* Many participants also described used dental therapy as a **s*tepping stone*** to get into their desired professions. This group included former dental therapists who did not obtain satisfactory results to get immediate acceptance into their desired degrees, such as dentistry or medicine. These participants had no interest in becoming dental therapists; hence, they obtained their dental therapy degrees and left the profession.

*I have always had an interest in a career in dentistry*, *but what happened when I completed school in matric*, *I was not able to get straight into dental school*. *I did not get into Wits*, *but then I got into the University of UDW (University of Durban Westville) for a degree in dental therapy*. *I could have gone to do n MSc*, *the BSc first*, *because I was also accepted at the University of Western Cape*, *but I thought that maybe starting with this BSc would throw me off and I wouldn’t ultimately get into my area of dentistry*.

*(DT 10*, *female)*

Some participants who deliberately chose to get a degree in dental therapy, did so to ***expand their scope***, upgrading their career from a two-year diploma in oral hygiene to a three-year degree in dental therapy. A fourth sub-theme related to a deliberate choice to enter dental therapy was ***stipends***. During the inception of dental therapy training, students received a monthly stipend for their training. This attracted some participants to the profession. Two participants mentioned that they enrolled in the profession to take advantage of the stipend. One explained: *“They were paying us; there was the stipend*, *a salary back in the days*, *which was the motivation*.*” (DT 6*, *male)*

Apart from those dental therapists who deliberately chose the profession, others ended up studying dental therapy through less intentional pathways, falling under our second theme, **unintentional**. Two sub-themes that we identified were: poor career guidance and applied late. ***Poor career guidance*** during high school played a significant role, leading some students to study dental therapy. Most of those interviewed got into the profession without knowledge of the scope of practice and some mentioned that they had no knowledge of the existence of the dental therapy course prior to their university admission. At the time of enrolment, most study participants could not differentiate between dental therapy and dentistry. Some participants mentioned that they only realised after enrolment that they were not doing dentistry, while others thought that they had to do dental therapy first in order to get to dentistry.

*I wanted dentistry*, *but I didn’t know firstly that you can do*, *you cannot do BDS (Bachelor of dental science) or dentistry*, *pure dentistry*. *But I was not really sure that what I was doing was not really BDS per se*. *So*, *I thought I was doing dentistry*, *but then I found out when I was doing*, *I think my second year*. *One of our lecturers told us that by the way you are not*, *you will not be dentists*, *you won’t be called doctors*. *So*, *I think it is poor career guidance*.

*(DT 11*, *male)*

Some people found themselves in the profession unintentionally because they ***applied late*** and therefore had to enrol for any available course in the institution. As one participant explained: *“I didn’t even know about it*, *I was just told that there’s only a space for dental therapy*.*” (DT 9*, *female)*

What was noteworthy about the interviews was that participants were passionate when talking about their previous role as dental therapists, referring to their skills and service delivery, although they were frustrated about the issues that led them to leave. Despite this passion, they still left because of the challenges they faced in South Africa. Many expressed a belief that it is the responsibility of government to support the sustainability of the profession. One participant explained:

*There’s a high need for it (dental therapy profession)*. *I still believe that there really is a high need for it*. *However*, *the support from government is not really there in terms of seeing that this profession is sustained in this economy*.*”*

*(DT 5*, *female)*.

### Theme 2: Extrinsic factors

Although most participants expressed their great enjoyment in the dental therapy profession, they also expressed dissatisfaction with various aspects thereof, which drove them away. The five extrinsic themes identified were poor remuneration, no career pathing, no job opportunities, no professional identity, and poor working conditions.

**Poor remuneration** was the most salient theme. When referring to poor remuneration, participants who worked in the public sector spoke about financial benefits such as salaries, policies (such as overtime), occupational specific dispensation (OSD), and retention allowances; while participants who worked in the private sector focused on unregulated salaries and low medical aid reimbursement fees.

All participants who worked in the public sector expressed their frustration with ***low salaries***. They felt that the remuneration policies were unfair and discriminatory against dental therapists, resulting in their decision to leave the profession. Dental therapists were not happy with earning the same salary as oral hygienists who held a two-year diploma while they held a three-year degree and had a broader scope of practice in the public sector.

*As much we deserve to be treated better and yet but it did not make sense to me because I (as an oral hygienist) did a diploma which was two years and as a dental therapist you will do three years*, *which is a degree*, *and they pay this person the same amount*. *I mean these two get paid the same amount*. *For me it did not make sense*.

*(DT 1*, *female)*.

Participants further expressed their frustration with the ***unfair implementation of remuneration policies*** in the public sector. They felt that they were treated differently as compared to other midlevel health professionals in the public sector such as physiotherapists, occupational therapists, and dieticians. They lamented exclusion from remuneration policies such as rural allowance, scarce skills allowance, and OSD policies, which benefited all other healthcare professionals, including midlevel health professionals.

*The fact that as a dental therapist*, *honestly*, *we are not really taken seriously*. *Most allied health workers get a rural allowance*. *We do not*. *So those kinds of things and then you start to wonder ukuthi kanti [where are we*?*]*, *where does the government really put us*.

*(DT 2*, *female)*

Like those in the public sector, salary was also a concern for those in the private sector, leading them to leave.

*I became discouraged*. *I felt a lot of discrimination*. *I did not have money*. *The money that we were paid then was not enough*. *I was getting something like R7 000 and then the money that I was getting I just could not cope with that*. *It was in the private sector*.

*(DT 8*, *female)*

Participants who were employed in the private sector felt that they were discriminated against because of the low salaries they earned. Most left the profession because they felt that the salary was too low to cater for their living expenses.

Study participants who worked independently in private practice also expressed their frustration with ***low medical aid reimbursement fees*** for the services they rendered. They compared themselves with dentists and felt that they should be paid the same fees for the same procedures performed, whether by the dentist or dental therapist. Their frustration was because both dentists and dental therapists use the same materials and the same equipment for those procedures, yet they were paid less.

*If you have to claim for a mask as a therapist*, *you get paid less*, *and then a dentist gets paid more for gloves and masks*, *which is not…ya*. *And an exam for a dentist is more expensive for a therapist*, *but all we did was examine the mouth*, *we have not done any…it is something that I can do…examining of the mouth*, *though a dentist does it*, *it is the same way the therapist will do it*.

*(DT 2*, *female)*

Participants mentioned that they were not involved in decision-making regarding the reimbursement fee structuring, which created discrepancies in their reimbursement because someone else was making decisions on their behalf.

**Lack of job opportunities** was another common theme. Whether they got into the profession deliberately or unintentionally, participants mentioned that the lack of job opportunities, especially in the public sector, contributed to their decision to leave. An altruistic desire to serve communities in the public sector was undermined by a lack of job opportunities, leading some to seek employment in the private sector. One participant said:

*I was forced to go and work in the private sector and even then*, *I was not prepared*. *I just did not like the private sector*. *I did not care much about money*. *I cared much about going back and serving the community*.

*(DT 8*, *female)*

In contrast, the participants who received funding from the Department of Health for their dental therapy degree did not struggle to find jobs in the public sector because of their contractual agreement to serve in the public sector following their graduation.

**Lack of professional identity** (which was not part of Hertzberg Two-Factor Theory) was another trending theme among former dental therapists. This common perception around lack of identity was expressed clearly by the following study participant:

*Dental therapy is a career struggling with its own identity*, *you are trying to be a dentist and you are running away from being an oral hygienist…*. *So*, *you fall somewhere within the cracks*. *You cannot be a dental therapist because the temptation is always very broad that you want to do some of the things that dentists do*.

*(DT 6*, *male)*

Another complaint was raised by a participant who had been practicing for more than 20 years. He bemoaned that people did not understand his scope of practice or role in the clinical space:

*We get tired of fighting and having to justify for everything that you do*, *or you know even to explain yourself*. *I mean I am 43 years now*. *If somebody asks me what you do dental therapists get for degree*, *it is more like you are asking who I am*, *something that I have been doing for 20 years and you are asking me after 20 years what I am doing or who am I*.

*(DT 11*, *male)*

Lack of professional identity affected their ***sense of belonging***. They felt this led to a lack of unity among the dental team because dental therapists were not treated as team members. One former dental therapist said,

*I think the reason why we (oral health team) are not improving is that as dentists and therapists and oral hygienists*, *we are not one family*. *We’re just under one umbrella*, *but the way we’re behaving we are not really fighting for each other*. *I think that’s the challenge*.

*(DT 1*, *female)*

***Lack of awareness about the dental therapy profession*** also contributed to feeling a lack of professional identity. Some study participants described how members of the public expected to find a dentist instead of a dental therapist. Gaining a sense of belonging and professional identity associated with dentistry or other professions was one reason these participants decided to leave the profession.

Most participants who worked in the public sector were demotivated by **poor working conditions** in their facilities, which was identified as another theme. Most cited ***lack of resources*** or poor maintenance of equipment, which limited them in terms of practicing their full scope. For instance, some reported only being able to do dental extractions due to lack of equipment to perform other procedures. Lack of support from the management contributed to their frustration, as they felt that dental services were not prioritised.

*The only problem now becomes when you are in the public sector and they don’t provide equipment and materials for you to do other things*. *It’s really tiring and also it gives you…it de-motivates you into enjoying your profession because now I didn’t really…for years all I did was extractions and probably you know give medication for abscesses and what not*, *you know*, *pain management*, *but that’s not all I went to school for*.

*(DT 2*, *Female)*

This lack of resources also affects most citizens who rely on public oral health services, as they do not have access to preventive services such as cleaning and restoring teeth.

### Theme 3: Intrinsic factors

Apart from the extrinsic factors that were related to job dissatisfaction, participants mentioned the lack of motivating factors, which pushed them away from the dental therapy profession. **Lack of career pathing** was a common theme. Former dental therapists felt that they were stagnant in their profession. They were frustrated by the fact that the dental therapy ***scope was limited***, and they could not further their studies within the profession, even if they liked what they did. Limited scope meant that they had to refer patients, in the process losing patient trust: *“Besides being a therapist*, *like for me*, *it’s like I was limited*. *Once I’m there I’m there*. *I’m stuck*, *and I cannot go anywhere” DT 4*, *female*.

Participants further explained that the dental therapy profession offered ***no career growth***, with no postgraduate program in dental therapy. Participants described the dental therapy profession as a dead-end career. Participants who worked in both public and private sector acknowledged that dental therapists have access to some postgraduate opportunities such as a Masters in dental therapy or public health; however, but they felt these ***postgraduate qualifications were not recognised*** and had no influence on either their scope of practice or remuneration.

*There are post-grad courses that we can do*, *but they’re not taking you to a level that you want*. *It’s like you’re still…you are just doing them for yourself but not really growth in terms of your career*.

*(DT 1*, *female)*

## Discussion

This study provides a summary of key challenges with attracting and retaining dental therapists in South Africa, from the perspective of former dental therapists. Most participants expressed that they never intended to be dental therapists; however, once qualified, they enjoyed the profession. These findings are similar to those of a South African longitudinal study conducted among the final-year health science students that reported that although 36.4% of participants were not studying towards their first-choice programs, 95.9% were happy to be serving and helping the community [22]. All participants appreciated the skills they acquired as dental therapists, and most of them still draw on those skills in their new professions or careers. They believed that dental therapy is fit for purpose, especially in the South African context with the high burden of unmet oral health treatment needs. Despite almost all participants expressing their love for dental therapy profession, they all left the profession. Most left due to intrinsic and extrinsic factors that led to job dissatisfaction as stipulated in the Hertzberg Two-Factor Theory, while others left because they never intended to practice as dental therapists.

The Hertzberg Two-Factor Theory helped to identify factors contributing to the attrition of dental therapists. Based on the findings, most factors that contributed to attrition were dissatisfaction with aspects of the health system; hence our recommendations were based on the house model, also known as the HRHSD framework [18]. The HRHSD, developed by the WHO, uses a visual representation of a house to depict the key components of a comprehensive and well-functioning health workforce [18, 23]. The HRHSD provides a holistic framework for analyzing and addressing challenges in human resources for health, emphasizing the interconnectedness of the different pillars and the need for a comprehensive approach to ensure a well-performing health workforce [18, 23]. By assessing and strengthening each pillar, policymakers and health system stakeholders can work towards improving the availability, competence, performance, and support of the health workforce, ultimately contributing to the delivery of quality healthcare services [18, 23]. Moreover, the HRHSD framework supports the development and implementation of targeted strategies and interventions to address these challenges. It emphasizes the importance of evidence-based decision-making and the use of data to inform workforce planning, policy formulation, and resource allocation. This ensures that interventions are tailored to specific workforce needs and have a higher likelihood of success [18, 23].

**Fig 2:**
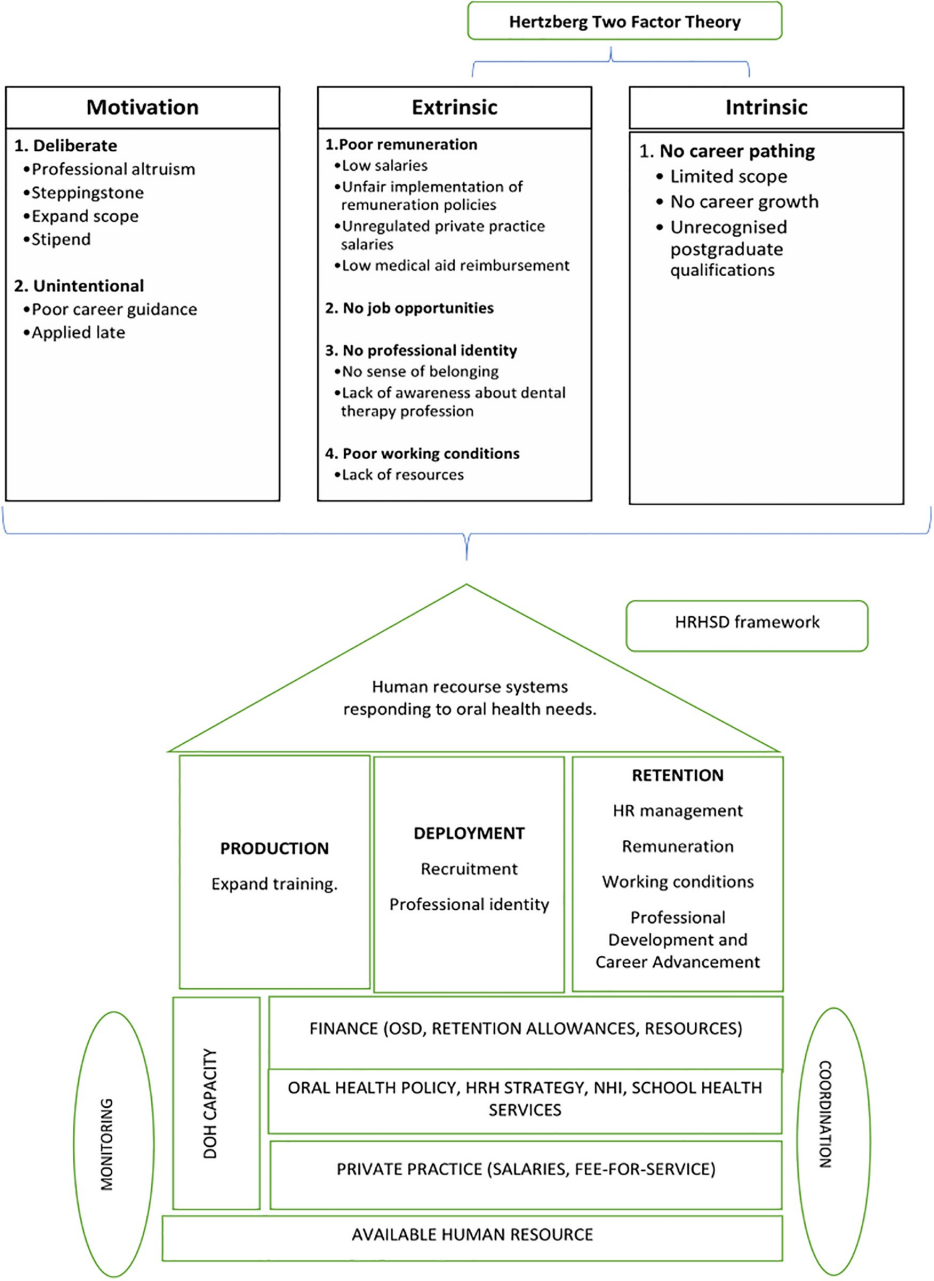
Current situation according to Hertzberg and recommended interventions according to the house model.

The two frameworks used in this study complement each other in this context of attrition of dental therapists, as the Herzberg’s Two-Factor Theory focuses on individual-level factors that impact job satisfaction and dissatisfaction [17], while the HRHSD framework provides a holistic approach to analyzing and addressing various dimensions of the health workforce, which includes production, deployment, and retention (see Fig 2).

Increasing the production of dental therapists is a key feature of our framework to address the issue of underproduction and low numbers caused by attrition of dental therapists. This would be not only economically beneficial [7], but it would also have the potential of improving the access to basic oral health services in South Africa. This is in line with the universal coverage that is intended in South Africa through the National Health Insurance (NHI) [24]. Increasing the annual production of dental therapists is not a new concept, it was first recommended by the South African Department of Health in 2006, although there is still resistance to respond to the recommendations to expand training to all dental schools [25]

In terms of production, the participants in our study provided unique insights into what led them into the dental therapy profession. With only two universities training dental therapists in South Africa [4], the finding that many dental therapists did not intend to enroll for the degree is concerning, particularly for other universities that may consider offering dental therapy training. Training institutions need to consider how to make this profession more attractive to prospective students to address the issue of underproduction, which was flagged in the recent 42-year review of the dental therapy profession in South Africa [14]. There is also a need to expand training to dental schools in other provinces to both increase the annual production and geographic coverage. However, increasing the production of dental therapists will not be viable if the extrinsic and intrinsic factors that were identified in our study are not addressed.

Increasing the production of dental therapists is interconnected with deployment, which requires their availability for equitable distribution within the healthcare system. Clear deployment strategies would address the perception that there is a lack of job opportunities, another reason for attrition in the study. As highlighted in the introduction, job opportunity concerns are common causes of attrition [16, 26] across all professions and industries. This is exacerbated by South Africa’s official unemployment rate of 44.4% in 2021, which is the highest in the world [27]. Deployment refers to the process of assigning dental therapists to specific positions or locations where their services are needed within the healthcare system. It involves strategically placing them in areas with a shortage of dental services or underserved populations. The deployment process should consider factors such as population needs, geographic distribution, oral health disparities, and availability of dental care facilitated on the oral health needs assessment of the population, which involves the analysis of the oral health status, disease burden, demographic factors, and healthcare service utilization patterns [8]. To achieve this there is a need for national oral health survey since the last national oral health survey was in 2002. Oral health needs assessment will help to determine the types and numbers of oral healthcare professionals required to address the population’s health needs. Based on the findings of the needs assessment, workforce planning should be conducted to identify the necessary oral health professionals required to provide comprehensive care, considering other factors such as skill mix, competencies, and specialties. Once the oral healthcare workforce requirements are identified, recruitment should follow.

One strategy to address attrition related to lack of recognition and professional identity would be to integrate dental therapists with other health programs. This has led to the perceived and perhaps real undermining and exclusion of dental therapists in many aspects of the health system; hence we recommend the integration of dental therapists with all health programs. Integration across health programs will support dental therapists’ sense of inclusion and could increase their visibility among other health care professionals. Deployment of dental therapists should go beyond the traditional dental care settings, with integration into all health programs, such as school-based health programs, maternal and child health, community/primary health care clinics, geriatric facilities, as well as and many other public health programs where there is an opportunity to promote oral health. Collaboration, coordination, and integration among different healthcare providers and programs is essential to improve access to basic oral health services.

Deployment has the potential to support retention strategies by providing dental therapists with opportunities for professional growth, skill development, and diverse work experiences. Strategic deployment that aligns with the career aspirations and developmental needs of dental therapists can contribute to their job satisfaction and commitment to staying within their profession. Our participants expressed dissatisfaction with the lack of career pathing, which they mentioned as one of the reasons they left the profession. Our findings are similar to that of a study conducted among allied healthcare workers, which reported lack of career structure in their professions, which also led to dissatisfaction and attrition [26]. Addressing the lack of career pathing among dental therapists requires a proactive approach from health systems. Hence, we recommend that key stakeholders, such as dental therapists, professional associations, academic institutions, employers, policy makers, oral health managers, and regulators should work together to identify areas of specialization or advanced skills that are in demand. These should be aligned with the evolving oral health needs of the country to identify specific areas where postgraduate training is needed. Based on the identified needs, academic institutions and regulators such as South African Qualifications Authority (SAQA) and HPCSA should develop postgraduate qualifications and funding for dental therapists. These curricula should be designed to provide advanced knowledge and skills in specialized areas of oral healthcare and research. By offering postgraduate opportunities for dental therapists, health systems can enhance their skills, knowledge, and career prospects, ultimately contributing to their job satisfaction and retention within the profession. This can be benchmarked from other countries such as Rwanda where the postgraduate training is linked to the scope expansion, these programs would not only benefit individual dental therapists, but also strengthen the overall capacity and quality of oral healthcare services.

To ensure the sustainability of the deployment strategy, continuous monitoring and evaluation of strategy is crucial to assess their performance, identify challenges, and make necessary adjustments. This will promote retention, which is a critical aspect of deployment. Other strategies that should be considered to promote retention include providing competitive salaries and benefits, offering career development opportunities, ensuring a supportive work environment, and recognizing and rewarding their contributions. All of these factors pushed our participants away from the dental therapy profession and have also been identified in other studies [16, 26].

Poor remuneration was the leading factor influencing the participants’ decision to leave the dental therapy profession. These results are similar to those reported in a study conducted among Australian oral health therapists, which mentioned low remuneration as a barrier to recruitment and retention [28]. In contrast, another study conducted among non-working dental therapists in Australia reported family commitments and career change as the leading reasons for attrition, while only 14% of participants cited poor pay [15]. Although South African dental therapists earn the same salary as other midlevel health workers such as physiotherapists, occupational therapists, and dieticians [29], participants were not happy with earning the same salary as oral hygienists who held a two-year diploma while they had a three-year degree. The same complaint was highlighted in a South African region study of dental therapists, where participants felt that dental therapists should be paid at a higher level than oral hygienists given their wider scope of practice and additional year of training [16]. The oral hygiene qualification has, however, been rectified in recent years and is now a three-year university degree [30].

While comparing themselves with other mid-level health workers in South Africa, participants mentioned the poor implementation of remuneration policies and exclusion from benefits or incentives enjoyed by all other midlevel health professionals, such as rural allowances and retention allowances. This felt exclusion might be, at least in part, due to poor implementation of policies. Implementation of remuneration policies such as OSD has been poor in South Africa [29, 31]. OSD was meant to provide career progression for all health professionals within their own professions [29], such as enabling a professional to move from a clinical position to a management position, such as a director. Addressing poor implementation of remuneration and retention policies within the context of human resources for health system development requires a thorough review of existing remuneration and retention policies to identify gaps or shortcomings in their implementation. To promote retention, part of that process should include an assessment of whether existing policies align with the needs and expectations of dental therapists. If gaps are identified, revisions may be required to ensure that the policies are perceived to be clear, transparent, and fair by dental therapists.

Participants who worked independently in the private sector left because they felt that they were discriminated against by medical aid. Their frustration that the reimbursement fees were lower than those of dentists for the same procedures that they performed was also highlighted in similar South African studies [17, 19]. While acknowledging their frustration, it is worth noting that dentists studied for five to six years while dental therapists studied for three years, which could be the reason why the medical aids recommend higher fees for dentists as compared to those of dental therapists. Additionally, the medical aid administrators only publish the recommended fees; hence, the practitioners (including dental therapists) are allowed to charge what they feel their services are worth, in agreement with the patient. For example, if the medical aid recommends that the consultation is worth R200, the practitioner can decide to charge a fee above the one recommended by the medical aid, but this must be reasonable and agreed upon by the patient prior to the consultation since the extra amount will be covered by the patient. We recommend that stakeholders who are responsible for decision regarding remuneration work collaboratively with dental therapists to ensure that remuneration policies and processes within the private sector are transparent, fair, and clearly communicated. Dental therapists need a clear understanding of how their remuneration is determined, including salary scales and fee for service. Collaboration and transparent policies will help build trust and confidence in the remuneration system, addressing potential dissatisfaction, and promoting retention.

Finally, our participants said poor working conditions led them to leave the dental therapy profession. Poor working conditions are not unique to dental therapists and have been reported by other health professionals as well [12]. A lack of resources such as equipment and support from managers also has been reported in studies conducted with dentists, with a South African study conducted in the Western Cape reporting that less than a third of dental facilities had the resources to offer the basic treatment package, with most clinics only offering extractions [12]. Poor working conditions, especially in public facilities, may hinder oral health professionals from practicing their scope, which our study suggests exacerbating attrition. This can be addressed through the allocation of sufficient funds and resources to support the implementation of oral health programs, which includes budgetary provisions for dental equipment and consumables.

In summary, we concur with the WHO observation that effective governance structures and policies are essential for the successful deployment and retention of human resources for health [8]. South Africa needs strong governance to ensure accountability, quality assurance, and ethical practice in increasing the production, deployment, and retention of dental therapists as part of its strategy to improve access to basic oral health services and achieve universal coverage. Deployment of dental therapists and retention are interconnected and mutually influential within the context of human resources for health system development. Addressing intrinsic and extrinsic factors, using a health system lens has the potential to enhance the efficiency of producing, deploying, and retaining dental therapists. This aligns with the objectives outlined in South Africa’s National Oral Health Policy, emphasizing preventive measures and oral health promotion [32]. Moreover, it resonates with the government’s recognition of dental therapists as a pivotal workforce in delivering fundamental oral health services [30] and supports the country’s aspirations of universal health coverage through the NHI, centered on reorienting healthcare services towards primary health care [24]. An increased retention rate of dental therapists would yield economic benefits for the nation attributed to the cost-effectiveness of training and employing dental therapists [7]. The cumulative effect of these improvements would contribute to a comprehensive enhancement of the oral healthcare system, promoting better oral health outcomes for the population.

The study acknowledges potential limitations, notably the risk of social desirability bias from participants as well as researcher bias from those authors with oral health backgrounds, particularly in dental therapy. Robust participant responses, as reported in the results, suggests that social desirability was not at play. Potential researcher bias was addressed through the involvement of co-coders without dental therapy backgrounds and oversight from a supervisor who is not a dental health professional, with no personal stake in the results. Another potential limitation is that the perspectives of some former dental therapists may have been missed. To reduce this risk, a multi-pronged sampling strategy was applied.

## Conclusion

This study revealed key factors influencing dental therapist attrition in South Africa, which are mostly related to job dissatisfaction due to lack of extrinsic and intrinsic factors mentioned in the Hertzberg Two-Factor Theory. Most of these factors can be addressed through targeted interventions that focus on specific issues related to production, deployment, and retention of dental therapists, including consideration of the cross-cutting areas of finance, policies, and the private sector. Addressing these factors is likely to improve production and retention of dental therapists, which will lead to improved access to basic oral health services. Ultimately, this improved access to oral health services may result in better oral health outcomes for the population in the country.

## Supporting information

S1 Checklist Consolidated criteria for reporting qualitative studies (COREQ): 32-item checklist. (PDF)

S1 File (DOCX)
