# Correction: Exploring reasons why South African dental therapists are leaving their profession: A theory-informed qualitative study

**DOI:** 10.1371/journal.pone.0300094

**Published:** 2024-03-05

**Authors:** Pumla Pamella Sodo, Yolanda Malele-Kolisa, Aneesa Moola, Veerasamy Yengopal, Simon Nemutandani, Sara Jewett

The third author’s name is spelled incorrectly. The correct name is: Aneesa Moolla. The correct citation is: Sodo PP, Malele-Kolisa Y, Moolla A, Yengopal V, Nemutandani S, Jewett S (2023) Exploring reasons why South African dental therapists are leaving their profession: A theory-informed qualitative study. PLoS ONE 18(10): e0293039. https://doi.org/10.1371/journal.pone.0293039
