# Supplementary material for: Exploring reasons why South African dental therapists are leaving their profession: A theory-informed qualitative study
Source: PLoS One. 2023 Oct 26;18(10):e0293039. doi: 10.1371/journal.pone.0293039 (PMC10602277; doi:10.1371/journal.pone.0293039)
Supplement: S1 File — (DOCX) [file pone.0293039.s002.docx]

ANNEXURE D: IN DEPTH INTERVIEW GUIDE FOR DENTAL THERAPISTS WHO LEFT THE PROFESSION

1. What motivated you to study for a degree in dental therapy?

2. How did your study experiences match your expectations in terms of preparing you for a career in dental therapy?

3. What was your experience as a dental therapist, if you ever practiced?

3.1 What are your views of working as a dental therapist in the private vs. public sector?

3.2. What are your views about the dental therapy profession in South Africa?

4. What influenced your decision to leave the dental therapy profession?

4.1 Employment opportunities?

4.2 Job satisfaction? Elaborate

4.3. Remuneration?

4.4. Working conditions?

4.5. Recognition?

4.6 Career pathing?

4.7 Scope?

Regulation?

1. To what extent does your current employment draw on skills you learned as a dental therapist?
2. What would make you go back to the profession if anything?
3. What can be done to improve dental therapy profession in South Africa?
4. What else would you like to share with me that we have not covered?

Thank you for your time and insights.
